# Catheter-associated urinary tract infection and urinary catheter utilization ratio over 9 years, and the impact of the COVID-19 pandemic on the incidence of infection in medical and surgical wards in a single facility in Western Qatar

**DOI:** 10.5339/qmj.2023.14

**Published:** 2023-05-02

**Authors:** Humberto Guanche Garcell, Jameela Al-Ajmi, Ariadna Villanueva Arias, Joji C Abraham, Angel M Felipe Garmendia, Tania M Fernandez Hernandez

**Affiliations:** ^1^Infection Control Department, The Cuban Hospital, Hamad Medical Corporation, Doha, Qatar. E-mail: humbertoguanchegarcell@yahoo.es ORCID: https://orcid.org/0000-0001-7279-0062; ^2^Corporate Infection Control Department, Hamad Medical Corporation, Doha, Qatar

**Keywords:** Urinary catheter, urinary tract infection, the incidence of infection, inpatient ward, acute care, COVID-19, Qatar

## Abstract

Introduction: Catheter-associated urinary tract infection (CAUTI) is a frequently reported healthcare-associated infection in critical and non-critical patients. Limited data are available about CAUTI incidence in non-critical patients. We aim to describe the incidence of CAUTI over 9 years and evaluate the impact of the pandemic on the incidence in non-critical acute care patients.

Methods: A retrospective observational study of CAUTI in medical-surgical and maternity wards was carried out at a public hospital in the west of the State of Qatar. Data collected included the annual CAUTI incidence (per 1,000 device days), urinary catheter utilization ratio (UC-UR), etiology, and antimicrobial resistance.

Results: 115,238 patient days and 6,681 urinary catheters (UC) days were recorded over the study period, and 9 and 4 CAUTI were confirmed in medical-surgical and maternity wards, respectively. The infection rate was 1.9 per 1,000 UC days, and the UC-UR was 0.06. The CAUTI rate was higher in medical-surgical wards over the COVID-19 period (2.4 × 1,000 UC days) in comparison with the non-COVID-19 period (1.7 × 1,000 UC days) (RR 1.46; 1.12–1.80). However, in the maternity ward, the result was 0 and 2.5 × 1,000 UC days during these periods, respectively. No differences were observed in the infection rate among periods for all patients (RR 1.06; 0.81–1.31). Multidrug-resistant organisms were identified in 7 patients, and non-multidrug-resistant in 6 cases.

Conclusion: The study findings describe a lower CAUTI risk over 9 years in non-critical acute care patients. The impact of COVID-19 on the CAUTI risk is mainly related to medical patients who had previously been admitted to critical care. The infection control program should consider these data as a benchmark for quality improvement.

## Introduction

Catheter-associated urinary tract infection (CAUTI) is among the most common healthcare-associated infections worldwide and is also associated with clinical complications and higher healthcare costs.^1,2^ The major incidence of CAUTI is reported in critically ill patients, whereas the risk in non-critical acute care in medical or surgical patients is lower.^3–6^ Catheterization duration in non-critical patients was the leading risk factor identified in various studies.^5,7^ Up to 69% of CAUTI could be prevented by implementing comprehensive programs.^8,9^

An increased incidence of healthcare-associated infections was described during the COVID-19 pandemic compared to the previous period, mainly for central line-associated bloodstream infection and ventilator-associated events, with a minor contribution from CAUTI.^8,9^ A recently published report describes the impact of COVID-19 on an increased CAUTI incidence of 43% (95% CI: 8–90%) in 148 healthcare-affiliated hospitals from American hospitals.^10^ In a single facility in Qatar, a higher CAUTI risk (3.25; 0.68–31.08) in critical patients was described during the COVID-19 pandemic, but limited published data are available about CAUTI incidence in non-critical patients.^11^

The Cuban Hospital, a public hospital facility and member of Hamad Medical Corporation (Doha, Qatar), provided COVID-19 patients with care from 2020 to 2021. Initially, a 75-bed public hospital was expanded to 385 beds later on, with the opening of tents as part of the national strategy to face the pandemic.^12^ The corporate infection control program (ICP) guides the facility’s infection control program.

Considering the evidence, a study aimed to describe the incidence of CAUTI in medical-surgical and maternity wards over 9 years and evaluate the impact of the pandemic upon non-critical acute care patients.

## Methods

This is a retrospective observational study of CAUTI at The Cuban Hospital. During the non-COVID-19 period (2014–2019, Jan–May 2022), the medical-surgical wards had a 54-bed capacity, and the maternity ward had a 10-bed capacity, and during the COVID-19 pandemic (2020–2021), the capacity was increased to 300 beds for medical-surgical, using tents and other hospital areas, while the maternity ward maintained a similar bed capacity.

Data were collected from the ICP records. The surveillance system was developed and set up by an infection control practitioner and a hospital epidemiologist. Due to potential variations in the infection control staff from 2017 to 2019, data were validated by reviewing patients’ medical records and reports from the microbiology laboratory. The data included the annual incidence of CAUTI (number of infections/number of the urinary catheter (UC) days per 1,000 device days) and UC-UR (number of UC days/number of patient days). Data on etiology and antimicrobial resistance were verified using the patient’s electronic medical records. CAUTI was confirmed using the Centers for Disease Control and Prevention (CDC, USA) definitions as per the corporate ICP.^13^ The corporate ICP includes the following measures: (1) infection control orientation for staff, (2) UC bundle embedded in the electronic medical records, and (3) surveillance of infection, including regular analysis and feedback conducted by a trained infection control team.

## Analysis

Descriptive statistical methods were used. CAUTI was presented as rates (per 1,000 device days) and percentile distribution. Statistical analyses were performed using the statistical package SPSS, version 22 (SPSS Inc., an IBM company, Chicago, IL). Relative risk (RR) ratios, 95% confidence intervals (CIs), and *p*-values were determined.

The two-tailed Student t-test was used to compare CAUTI incidence during the pre-COVID-19 and COVID-19. A *p*-value (two-tailed) of ≤0.05 was considered significant.

## Ethical Approval

The study was approved by the Institutional Review Board and the Medical Research Center (Hamad Medical Corporation, Doha, Qatar) (MRC-01-22-409). The exemption from obtaining patient consent was granted because the research will collect existing data, and patient-level information is recorded using identifiers linked to patients.

## Results

**Figure 1.**
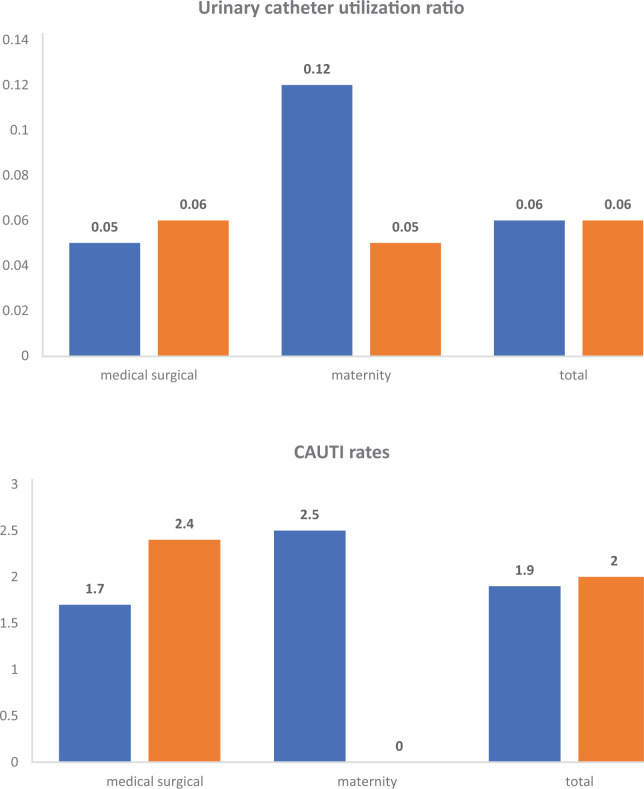
UC-UR and CAUTI rates (per 1,000 device days) during 2014–2019, 2022 (non-COVID-19 period), and 2020–2021 (COVID-19 period) in medical-surgical and maternity wards.

115,238 patient days and 6,681 UC days were recorded over the study period, and 9 and 4 CAUTI were confirmed in medical-surgical and maternity wards, respectively (Table I). The CAUTI rate was 1.9 per 1,000 UC days, and the UC-UR was 0.06. A total of 3 CAUTIs were confirmed during the COVID-19 period out of 13 CAUTIs over the 9-year study period. The infection rate in the medical-surgical wards was 1.7 and 2.4 × 1,000 UC days (RR 1.46; 1.12–1.80), and for the maternity ward, it was 2.5 and 0 × 1,000 UC days during non-COVID-19 and COVID-19 periods, respectively. No statistically significant differences in infection rates were observed among periods for all patients (RR 1.06; 0.81–1.31) (*p* > 0.05). In medical-surgical wards, the UC-UR was higher during the COVID-19 period, while in the maternity ward, it was lower during the COVID-19 period in comparison to the non-COVID-19 period (Figure 1).

The etiology of infection was related to multidrug-resistant organisms (MDRO) in 7 patients (*Escherichia coli* (5 isolates), *Klebsiella pneumonia* (1 isolate), and *Pseudomonas aeruginosa* (1 isolate). In addition, 6 cases with non-multidrug-resistant organisms, including 2 *Pseudomonas aeruginosa* and one each of *Citrobacter freundii*, *Enterobacter cloacae*, *Serratia marcences*, and *Enterococcus faecalis*, respectively, were also identified.

## Discussion

The study has described the low incidence of CAUTI over nine years in medical-surgical and maternity patients in a single hospital facility in the State of Qatar, with an increased risk of infection, focused on medical patients.

The incidence of infection was high for medical-surgical and maternity wards (1.9 and 2.2 per 1,000 UC days) compared with 2013 CDC data (1.3 and 0.2 per 1,000 UC days) and the data reported by a community hospital network in North Carolina (USA).^6,14^ Other studies described a higher incidence of CAUTI in cesarean section and in a general hospital.^3,15^ Studies summarized by Yu et al. described higher incidence rates in medical-surgical non-critical patients and the effectiveness of multifaceted, evidence-based interventions for CAUTI prevention.^16^ These include local adoption of guidelines, monitoring and feedback on infections and prevention practices, and education and training, among others.^16^

During COVID-19, the observed changes in UC use and associated infections were reported about selected patient factors and catheter care practices in a pandemic environment. In medical-surgical patients, the use of catheters was linked to previously critical patients, mainly medical patients with severe COVID-19 pneumonia who required respiratory support. In these patients, several factors determined the higher CAUTI risk during the pandemic, including (1) low staff coverage mainly during pandemic peaks, (2) limited infection control training of newly hired staff, (3) delay of catheter removal, and (4) low compliance with hand hygiene. The staffing and resources-related issues (coverage and training) constituted a remarkable problem worldwide during the pandemic, as well as compliance with infection control practices.^12,17^ The timely removal of UC contributes to CAUTI risk reduction, and the delay of removal was identified as a significant factor during the pandemic in our setting.^18–20^ In cesarean section patients, UC use was limited to the perioperative period, keeping the same practices for catheter care as during the pre-COVID-19 period.

However, only a few published studies describing the increasing incidence of CAUTI refer to a mixed population of critical and non-critical patients, which limits the comparison with this research.^9,21–23^ Fakih et al., with data from 78 US hospitals, described an improvement in the infection rate in non-critical patients,^24^ and Wee et al., in a general hospital in Singapore, show similar rates (1.8 per 1,000 UC days) during the COVID-19 pandemic than during the pre-pandemic period. The etiology of infections detected was similar to other reports, but the burden of MDRO was not evaluated because of the large number of isolates.^25^

The study has a few limitations, including a retrospective observational design and single-center data collection, which limits data comparison with groups of similar characteristics. The impact of data collection by various infection control staff (mainly for 2017–2019) was minimized by accurate data validation. The study’s main strength is the use of a standardized surveillance system and procedures guided by a corporate ICP. Thus, as a single-center study, the data provide evidence of the priority of infection prevention at the facility and corporate levels.

## Conclusion

Our findings describe a lower CAUTI risk over 9 years in non-critical-acute-care patients and an increased infection rate during the COVID-19 pandemic, mainly in medical patients transferred from critical care. The CAUTI prevention program should be reviewed to face future pandemics, with particular reference to compliance with infection control practices. The ICP must consider this data as a benchmark for quality improvement initiatives.

### Acknowledgment

We thank Alexis Gonzalez Velázquez for proofreading the manuscript.

### Authors contributions

Study design: HGG. Data acquisition: HGG and AVA. Data analysis: HGG, JAA, AVA, JCA, AMFG, and TMFH. Manuscript writing: HGG. Critical review and primary scientific input: JAA, AVA, JCA, AMFG, and TMFH.

### Conflicts of interest

All authors declare that they have no conflicts of interest.

### Funding

The authors received no financial support for this research.

## Figures and Tables

**Table 1.** Pooled means and key percentiles of the distribution of CAUTI and UC-UR in medical-surgical and maternity wards at The Cuban Hospital between 2014 and 2022.

**Infection rate**	**Infections**	**Device days**	**Pooled mean**	**10%**	**25%**	**50% (median)**	**75%**	**90%**
Medical surgical	9	4,836	1.9	0.00	0.00	1.22	2.76	–
Maternity	4	1,845	2.2	0.00	0.00	0.00	3.13	–
Total	13	6,681	1.9	0.00	0.00	1.22	3.14	–
Device utilization	Patients days	Device days	Pooled mean	10%	25%	50% (median)	75%	90%
Medical surgical	97,218	4,836	0.05	0.02	0.04	0.05	0.06	–
Maternity	18,020	1,845	0.10	0.04	0.08	0.12	0.13	–
Total	1,15,238	6,681	0.06	0.03	0.04	0.06	0.06	–
